# Immune checkpoint inhibitors for patients with mismatch repair deficient or microsatellite instability-high advanced cancers: a meta-analysis of phase I–III clinical trials

**DOI:** 10.1097/JS9.0000000000002007

**Published:** 2024-08-14

**Authors:** Wei Wang, Zubing Mei, Yajie Chen, Jian Jiang, Yanli Qu, Keyoumu Saifuding, Ning Zhou, Gilisihan Bulibu, Yong Tang, Xinyu Zhai, Zhi Jiang

**Affiliations:** a Department of Digestive Internal Medicine, The Affiliated Tumor Hospital of Xinjiang Medical University, Urumqi, Xin Jiang Province; b Department of Anorectal Surgery, Shuguang Hospital, Shanghai University of Traditional Chinese Medicine; c Anorectal Disease Institute of Shuguang Hospital; d Department of Urology, Shuguang Hospital, Shanghai University of Traditional Chinese Medicine Shanghai; e Department of Perioperative Research Center of Chinese Medicine, the Second Affiliated Hospital of Guangzhou University of Chinese Medicine; f Guangdong Provincial Key Laboratory of Clinical Research on Traditional Chinese Medicine Syndrome, Guangzhou, People’s Republic of China

**Keywords:** advanced cancers, clinical trials, efficacy, immune checkpoint inhibitors, microsatellite instability-high, mismatch repair deficient, personalized immunotherapy

## Abstract

**Background:**

Mismatch repair deficient (dMMR) and microsatellite instability-high (MSI-H) cancers are associated with an increased number of somatic mutations, which can render tumors more susceptible to immune checkpoint blockade. However, a comprehensive evaluation of the efficacy profile of immune checkpoint inhibitors in this patient population across multiple cancer types is lacking. This study aims to address this knowledge gap by synthesizing data from phase I–III clinical trials.

**Methods:**

A systematic search was conducted in PubMed, Embase, the Cochrane Central Register of Controlled Trials, and Google Scholar from inception until June 2024. Eligible studies included randomized controlled trials (RCTs), nonrandomized comparative studies, and single-arm trials investigating immune checkpoint inhibitors in patients with dMMR/MSI-H advanced cancers. The primary outcome was objective response rate (ORR), and the secondary outcomes included disease control rate (DCR), 1-year, 2-year, and 3-year overall survival (OS) and progression-free survival (PFS) rates. Subgroup analyses were conducted for the primary outcome stratified by major study characteristics.

**Results:**

Of the 10 802 identified studies, 19 trials in 25 studies totaling 2052 participants met the inclusion criteria and were included in the meta-analysis. The pooled ORR was 41.7% (95% CI, 35.7–47.7%). The pooled DCR was 68.9% (95% CI, 62.2–75.7%). The pooled 12-month, 24-month, and 36-month OS rates were 29.1% (95% CI, 19.9–38.3%), 35.8% (95% CI, 23.6–48.0%), and 35.8% (95% CI, 23.6–48.0%), respectively. The pooled 12-month, 24-month, and 36-month PFS rates were 46.4% (95% CI, 39.1–53.8%), 67.0% (95% CI, 55.2–78.8%), and 63.1% (95% CI, 37.3–88.9%), respectively.

**Conclusions:**

The study establishes the therapeutic potential of immune checkpoint inhibitors in dMMR/MSI-H advanced cancers, highlighting the importance of MSI status in this context. Further, head-to-head comparisons are needed to conclusively determine MSI’s predictive power relative to proficient mismatch repair/microsatellite stable (pMMR/MSS) tumors.

## Introduction

Highlights

- Meta-analysis confirms immune checkpoint inhibitors’ efficacy in dMMR/MSI-H cancers, with high response rates and durable survival outcomes, and emphasizes MSI as a critical immunotherapy predictor.
- The study reinforces MSI status as a pivotal predictor for immunotherapy responsiveness, emphasizing its integration into diagnostic algorithms.
- This study encompasses a broad spectrum of cancer types, strengthening evidence for ICI efficacy across the dMMR/MSI-H cohort.
- Findings advocate for personalized immunotherapy strategies based on genetic profiling, shaping future cancer care.

The burgeoning field of immuno-oncology has revolutionized the prognostic landscape for patients with advanced cancers, predominantly through the advent of immune checkpoint inhibitors (ICIs)^1–4^. These agents, which modulate the inhibitory pathways in T cells to enhance immune responses against cancer cells, have emerged as a cornerstone in the treatment of a myriad of advanced malignancies^5^. Despite their success, the epidemiology of advanced tumors presents a significant hurdle to the universal applicability of ICIs due to the vast heterogeneity of tumor biology and immune responsiveness. The treatment bottlenecks for advanced cancers are multifaceted, including the development of resistance to conventional therapies and limited durability of responses^6,7^.

Immunotherapy with ICIs, targeting CTLA-4, PD-1, and PD-L1, has been noted for its role in improving survival in various advanced cancers, such as melanoma and non-small cell lung cancer^8^. However, the impressive responses observed in a fraction of patients have necessitated an in-depth analysis to identify predictive biomarkers of efficacy to tailor immunotherapeutic approaches. The identification of key biomarkers such as mismatch repair deficiency (dMMR) or microsatellite instability-high (MSI-H) has carved out a subset of patients who display an enhanced treatment response due to the unique tumor mutational landscape^9,10^.

MSI-H is a hallmark of tumors with defective dMMR, leading to an accumulation of insertions, deletions, and substitutions at repetitive DNA sequences. This genomic instability fosters a unique microenvironment characterized by a high mutational load and the expression of a broad range of neoantigens, which, in turn, stimulates an augmented immune response against the tumor. Indeed, the immunogenicity of MSI-H tumors is significantly elevated compared to microsatellite stable (MSS) tumors, rendering them more recognizable to the host’s immune system^11^. This distinctive profile has sparked extensive interest in the potential for ICIs to exploit this heightened immunogenicity; preliminary studies have indeed hinted at exceptional responses to ICIs in this subset of patients^12–15^.

However, while the promise of ICIs in dMMR/MSI-H cancers is alluring, the literature remains peppered with inconsistencies and uncertainties^16–19^. On the one hand, there are accounts of remarkable clinical benefits, including durable responses and even complete remissions in a subset of patients^10,15^. Conversely, reports of hyperprogressive disease (HPD), where tumors grow at an accelerated rate after initiation of ICI therapy^20^, have raised concerns and highlighted the need for a nuanced understanding of ICI effects in this population. The dichotomy of outcomes underscores the complex interplay between tumor biology, immune surveillance, and therapeutic intervention and underscores the necessity for a more comprehensive appraisal of ICI efficacy.

Given these disparities and the pressing need for clarity in clinical decision-making, our meta-analysis takes a pivotal step forward. By consolidating evidence from phase I–III clinical trials focusing on ICIs in advanced dMMR/MSI-H cancers, we aim to navigate the convoluted landscape of therapeutic outcomes. Our approach is not only to quantify the aggregate response rates but also to discern patterns that might elucidate the underlying mechanisms connecting dMMR/MSI-H status with ICI response. By synthesizing the available data, we aspire to clarify whether the mutational burden associated with MSI-H status truly translates into improved clinical outcomes across various cancer types. Ultimately, this work seeks to refine the predictive value of MSI status as a biomarker for ICI response, thereby enriching the discourse on personalized immunotherapy and guiding clinical practice in the realm of advanced oncological care.

## Methods

### Study registration

This study was prospectively registered with the International Prospective Register of Systematic Reviews (PROSPERO) (with the registration number CRD42024504419). The protocol encompasses a clear articulation of the objectives, inclusion criteria, interventions, outcome measures, and planned statistical analyses in alignment with the Preferred Reporting Items for Systematic Reviews and Meta-Analyses (PRISMA) guidelines (Supplemental Digital Content 1, http://links.lww.com/JS9/D329, Supplemental Digital Content 2, http://links.lww.com/JS9/D330). The detailed protocol of the study can be accessed via the website https://www.crd.york.ac.uk/prospero/display_record.php?RecordID=504419.

### Search strategy

In adherence to the PRISMA guidelines and Cochrane Handbook^21,22^, we enhanced our systematic search strategy, initially conducted up to September 2023, by implementing a snowballing technique. This involved a meticulous review of the reference lists from all included studies and relevant systematic reviews. Additionally, we reiterated our search across all databases – PubMed, Embase, the Cochrane Central Register of Controlled Trials (CENTRAL), and Google Scholar – as of June 2024, ensuring the incorporation of the latest evidence into our meta-analysis.

Keywords and medical subject headings (MeSH) terms related to cancer, immune checkpoint inhibitors, mismatch repair deficiency, and microsatellite instability were used, individually and in various combinations, to maximize the retrieval of relevant studies. The search strategy was peer-reviewed by an expert in medical information retrieval to ensure comprehensiveness, which is presented in the Supplementary Material (Supplemental Digital Content 3, http://links.lww.com/JS9/D331). No limitations were placed on language, publication status, or publication date to encompass the entirety of the available literature.

### Selection criteria

The inclusion criteria for this pooled analysis were meticulously designed to capture phase I–III clinical trials that evaluated the use of immune checkpoint inhibitors in patients diagnosed with advanced cancers exhibiting dMMR or MSI-H characteristics. Exclusion criteria were delineated to exclude studies that did not clearly demarcate patient groups based on these genetic markers, trials that did not have clearly defined clinical outcomes of interest [e.g., objective response rate (ORR), disease control rate (DCR), overall survival (OS), and progression-free survival (PFS)], and any non-clinical trial publications, such as editorials, commentaries, and reviews, to mitigate the inclusion of indirect evidence. Two independent reviewers screened titles and abstracts yielded by the search strategy to identify studies potentially meeting the inclusion criteria. Subsequently, the same reviewers assessed full-text articles for final inclusion. Potential discrepancies were resolved through discussion or the involvement of a third reviewer, maintaining methodological rigor and concordance with our predetermined selection protocol.

### Data extraction

Data extraction was executed from all studies, fulfilling the inclusion criteria. A standardized data extraction form was developed, and a pilot test was performed on a sample of included studies to ensure the comprehensive capture of relevant information. The data extracted included study characteristics and patient demographics, which are pivotal for understanding the context and generalizability of the study findings.

Key variables and corresponding data extracted comprised first author, year of publication, name of the trial, geographic location of the trial, study design, sample size, distribution of cancer types (expressed in percentage), median age of participants, percentage of male participants, percentage of patients who had received prior treatments, type of immune checkpoint inhibitor administered, median follow-up period, clinical outcomes, including ORR, PFS, and OS.

Two independent researchers performed the data extraction, with disagreements resolved by consensus or by arbitration with a third reviewer. To ensure the reliability of data extraction, a subset of the included studies was cross-checked by both reviewers.

### Study quality assessment and grading evidence

The methodological quality and risk of bias in randomized controlled trials (RCTs) were assessed using the Cochrane Collaboration’s Risk of Bias Tool (ROB)^21^. Each RCT was examined for biases related to selection, performance, detection, attrition, reporting, and other biases. Nonrandomized studies were evaluated using the Methodological Index for Non-Randomized Studies (MINORS)^23^, which provides a comprehensive tool for assessing the methodological quality of comparative and non-comparative observational studies. The quality assessment was conducted independently by two reviewers. Disagreements were reconciled through discussion or with the input of a third reviewer to reach a consensus.

Additionally, to systematically evaluate the quality of evidence from our pooled analyses, we employed the GRADE (Grading of Recommendations Assessment, Development, and Evaluation) approach. This method allowed us to assess the certainty of the evidence for each outcome (ORR, DCR, 1-year, 2-year, and 3-year OS and PFS) based on factors such as study design, consistency of results, the precision of effect estimates, and potential publication bias. The quality of evidence was graded as high, moderate, low, or very low.

To ascertain the precision of our study assessments and mitigate subjective bias, we estimated inter-rater reliability using Cohen’s kappa coefficient. The kappa statistic was derived from a random sample of studies assessed independently by two researchers, providing a robust measure of concordance on study inclusion, data extraction, risk of bias, and grading evidence evaluations.

### Statistical analysis

We used Stata 12 software for all statistical analyses. The main outcome measures, including ORR and DCR, were pooled using a random-effects model, given the anticipatory heterogeneity inherent among the included studies. ORR and DCR were analyzed as proportions within the included studies and synthesized accordingly. The analysis extended to OS and PFS, which were considered not as continuous time-to-event data but as survival rates at specific time points – namely at 12, 24, and 36 months. The pooled estimates for the survival rates were calculated using the DerSimonian and Laird random-effects model, which assumes that the true underlying effect varies between studies^24^. Given the likely clinical and methodological diversity among the trials analyzed, we prespecified the use of a random-effects model for all outcomes to provide a more conservative estimate that acknowledges and incorporates this potential between-study heterogeneity. Heterogeneity among the included studies was quantified using the *I* ^2^ statistic^25^, with an *I* ^2^ value over 50% indicating substantial heterogeneity. Subgroup analyses were employed to identify determinants of efficacy and sources of heterogeneity among the varied trial populations. We investigated the influence of distinct factors including sample size, study year, geographic location, study design, cancer type, patient demographics (age and sex), extent of pretreatment, type of immune checkpoint inhibitor, and follow-up period. The possibility of publication bias was assessed through a two-pronged approach: careful visual inspection of funnel plots for asymmetry and the application of Egger’s test for statistical evidence of bias^26^. We maintained a pre-stipulated threshold *P*-value of less than 0.10 as indicative of potential publication bias. Sensitivity analyses were undertaken to verify the strength of our pooled results. We conducted sensitivity analyses to ascertain the stability of results under the omission of outlier studies that could influence the heterogeneity observed. For all the statistical tests performed, a significance level was set at a *P*-value less than 0.05.

## Results

### Literature search results

**Figure 1:**
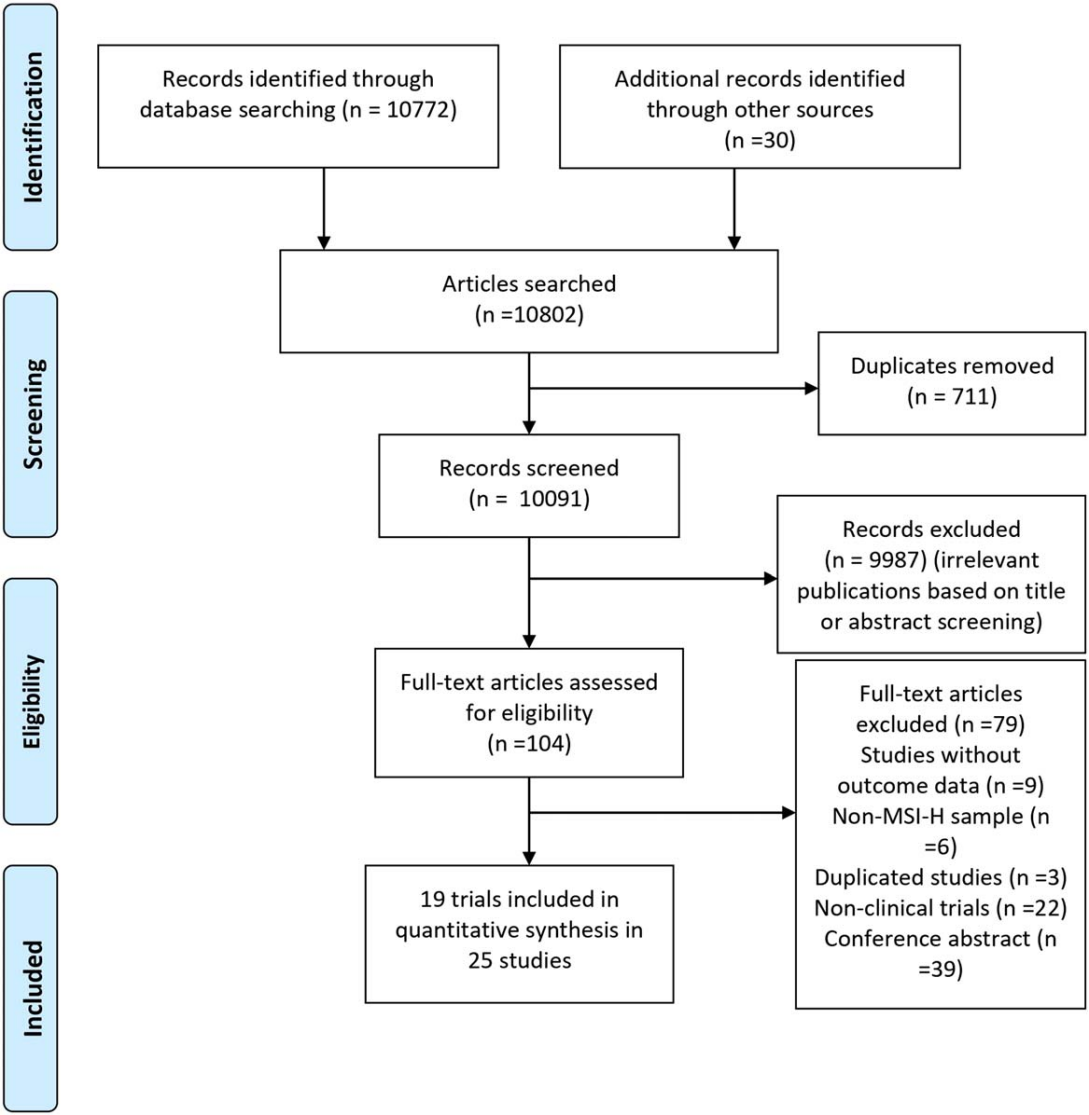
Flow diagram of the study selection.

Our comprehensive literature search initially identified 10 802 records. Upon screening titles and abstracts, 104 studies were assessed for eligibility and retrieved for full-text review thoroughly. Out of these, we selected 25 studies from 19 trials for inclusion in the pooled analysis^27–51^. Reasons for exclusion of studies consisted of the following: 9 studies did not provide specific data on ORR, DCR, OS, or PFS; 6 studies did not include MSI-H sample; 3 duplicate studies and 22 non-clinical trials; and 39 were conferences abstracts without detailed data for analysis. The detailed selection process for this pooled analysis is illustratively presented in Figure 1.

### Characteristics of the identified studies

In our meta-analysis, data were retrieved from a total of 2052 individuals across 25 studies from 19 trials (Table 1). The trials spanned over six continents, primarily focusing on four cancer types. Six studies assessed patients with colorectal cancer (CRC), 6 analyzed endometrial cancer, 11 studies incorporated multiple cancer types including but not limited to colorectal cancer and gastroesophageal cancers. The median age ranged from 50 to 69 years, with male percent making up from 0% to 100% of participants. Studies varied in size, with patient numbers ranging from 7 to 327, and subjects from 76% of the included studies had received previous treatments. Treatment interventions primarily centered around PD-1 inhibitors, utilized in 11 of the studies, while PD-L1 inhibitors were the subject of 9 studies. The remaining 2 studies provided insights into combination therapies involving both PD-1 and CTLA-4 inhibitors, 2 analyzed combination therapies involving both PD-L1 and CTLA-4 inhibitors,1 analyzed combination therapies involving both PD-1 and anti-TIM-3 antibody inhibitors. The median follow-up extended from 5.6 to 44.5 months.

**Table 1:** Basic characteristic and design of the included studies.

Reference	Year	Trial name	Location	Design	Sample size	Cancer types (%)	
Westin^48^	2024	DUO-E/GOG-3041/ENGOT-EN10 (NCT04269200)	Europe, North and South America, Asia Pacific	Randomized, double-blind, placebo-controlled multicenter phase III trial	94	Endometrial cancer	
Mirza^47^	2023	NCT03981796	USA	Phase 3, global, double-blind, randomized, placebo-controlled trial	53	Endometrial cancer	
Taïeb^49^	2023	SAMCO-PRODIGE 54 (NCT03186326)	France	A national open-label phase 2 randomized clinical trial	61	Metastatic colorectal cancer	
Eskander^46^	2023	NRG-GY018 (NCT03914612)	Canada, the United States, Japan, and South Korea	International, double-blind, placebo-controlled, randomized, phase 3 trial,	112	Endometrial cancer	
André^50^	2023	GARNET (NCT02715284)	Canada, Europe, USA	Multicenter, single-group, open-label, phase 1	327	Endometrial cancer (43.1), colorectal cancer (32.1), gastric and gastroesophageal junction cancer (21), small-intestinal cancer (5.8), small-intestinal cancer (3.4), pancreatic carcinoma (3.4), biliary neoplasm (10), ovarian cancer (7), other (including adrenal cortical carcinoma, unknown origin cancer, esophageal cancer, mesothelioma, breast cancer, malignant neoplasm of the female genitals, renal cell carcinoma, sarcoma, and thymic tumor) (13)	
André^30^	2023	NEONIPIGA	France	Single-arm, multicenter academic phase II study	32	Gastric or esophagogastric junction adenocarcinoma	
Chen^29^	2023	NCT04304209	China	Open-label, single-center phase 2 study	16	Advancer rectal cancer	
Rubinstein^27^	2023	NCT03015129	USA	Single-center, randomized, open-label, phase 2 study	38	Endometrial cancer or endometrial carcinosarcoma	
Geurts^28^	2023	Drug Rediscovery Protocol (DRUP)	Netherlands	Prospective, multicenter, nonrandomized clinical umbrella and basket trial	26	Colorectal cancer (31), endometrial cancer (12), small intestine cancer (12), stomach cancer (12), bile duct cancer (12), breast cancer (8), pancreatic cancer (4), prostate cancer (4), neuroendocrine cancer (4), glioblastoma (4)	
Qin^31^	2022	NCT03941574	China	Single-arm, open-label, phase II trial	68	Previously treated unresectable or metastatic MSI-H/dMMR solid tumors	
O’Malley^34^	2022	KEYNOTE-158	21 countries in Africa, the Americas, Asia, and Europe	Open-label, multicohort, phase II study	79	MSI-H/dMMR endometrial cancer	
Bellone^39^	2022	NCT02899793	USA	Single-arm, open-label, phase 2 pilot study	24	Recurrent lynch-like versus sporadic endometrial cancers	
Cercek^38^	2022	NCT04165772	USA	Single-group, prospective phase 2 study	16	Mismatch repair deficient, locally advanced rectal cancer	
Oaknin^32^	2022	GARNET study (NCT02715284)	Canada, Europe, USA	Multicenter, single-arm, open-label, phase I trial	106	Advanced and recurrent endometrial cancer (100)	
Diaz^37^	2022	KEYNOTE-177 (NCT02563002)	Asia, Western Europe, North America, rest of the world	International, randomized, open-label, phase 3 study	153	Metastatic colorectal cancer (100)	
Kwon^51^	2021		Korea	Phase II trial	18	Gastric cancer	
Li^40^	2021	NCT03667170	China	Multicenter, open-label, single-arm phase 2 study	103	Colorectal cancer (61), gastric/gastroesophageal junction cancer (6.5), endometrial cancer (6), hepatocellular cancer (2), hepatocholangiocarcinoma (2), bladder cancer (1), cervical cancer (1), cholangiocarcinoma (1), esophageal cancer (1), non-small cell lung cancer (1), osteosarcoma (1), prostate cancer (1), renal pelvic carcinoma (1), urothelial carcinoma (1), uterine sarcoma (1)	
Hollebecque^41^	2021	PACT (NCT02791334)	Canada, Spain, France, Belgium, South Korea, Chinese Taiwan	Multicenter, phase Ia/1b trial	82	Colorectal (47.6), endometrial (17), gastric (8.5), small intestine (8.5), esophageal (2.4), pancreatic (2.4), sarcoma (2.4), ovarian (1.2), cholangiocarcinoma (1.2), prostate adenocarcinoma (1.2), adrenal carcinoma (1.2), adenocarcinoma of the unknown primary site (3.7)	
Chao^42^ (NCT02335411)	2021	KEYNOTE-059 (NCT02335411)	Australia, Europe, North America, Asia, other regions	Multicohort, open-label, nonrandomized, phase 2 study	7	Advanced gastric/gastroesophageal junction cancer (NR)	
Chao^42^ (KEYNOTE-061)	2021	KEYNOTE-061 (NCT02370498)	Australia, Europe, North America, Asia, other regions	Multicenter, randomized, open-label, phase 3 study	15	Advanced gastric/gastroesophageal junction cancer (NR)	
Chao^42^ (KEYNOTE-062)	2021	KEYNOTE-062 (NCT02494583)	Australia, Europe, North America, Asia, other regions	Randomized, controlled, partially blinded interventional, first-line treatment, phase 3 trial	14	Advanced gastric/gastroesophageal junction cancer (NR)	
Marabelle^52^	2020	KEYNOTE-158 (NCT02628067)	21 countries in Africa, the Americas, Asia, and Europe	Nonrandomized, open-label, multisite phase II study	233	Endometrial, gastric, cholangiocarcinoma, pancreatic, small intestine, ovarian, brain, sarcoma, neuroendocrine tumor, cervical, prostate, adrenocortical, breast, thyroid, urothelial, mesothelioma, small cell lung cancer, renal (NR)	
Le (Cohort A)^53^	2020	KEYNOTE-164 (NCT02460198)	Asian, Black/African American, White	International, phase 2, open-label, nonrandomized, multicenter	61	Metastatic colorectal cancer (100)	
Le (Cohort B)^53^	2020	KEYNOTE-164 (NCT02460198)	Asian, Black/African American, White	International, phase 2, open-label, nonrandomized, multicenter	63	Metastatic colorectal cancer (100)	
Cohen^43^	2020	GERCOR NIPICOL (NCT03350126)	France	Multicenter, single-arm, open-label, phase 2 study	57	Metastatic colorectal cancer (100)	
Azad^54^	2020	NCI-MATCH (EAY131) subprotocol Z1D (NCT02465060)	White, Black, Asian, Hispanic, native American	Open-label, single-arm, phase 2 trial	42	Endometrioid endometrial adenocarcinoma (31), prostate adenocarcinoma (12), uterine carcinosarcoma (9.5), adenocarcinoma of the esophagus/esophagogastric junction (7), cholangiocarcinoma (7), ductal carcinoma of the breast (7), pancreatic neuroendocrine carcinoma (2.4)	
Konstantinopoulos^55^	2019	NCT02912572	White, Black, Asian	Nonrandomized, two-cohort, phase 2 study	33	Metastatic endometrial cancer (100)	
Michael^44^	2018	CheckMate-142	Australia, Belgium, Canada, France, Ireland, Italy, Spain, and the USA	Multicenter, open-label, phase 2 trial	119	Metastatic endometrial cancer	
Author	Year	Age, median (year)	Male (%)	Pretreatment percent (%)	ICI type	Median follow-up period (months)	Outcomes
Westin^48^	2024	65	100	0	Dostarlimab (PD-L1)	16	PFS
Mirza^47^	2023	66	100	0	Dostarlimab (PD-L1)	33	PFS, OS
Taïeb^49^	2023	65	46.7	53.3	Avelumab (PD-L1)	33.3	ORR, DCR, PFS, OS
Eskander^46^	2023	67	0	35.7	Pembrolizumab (PD‑1)	12	PFS
André^50^ (GARNET)	2023	63	28.1	100	Dostarlimab (PD-L1)	27.7	DOR, PFS, OS
André^30^ (NEONIPIGA)	2023	65	72	0	Ipilimumab (CTLA-4)+Nivolumab (PD-L1)	14.9	EFS, OS
Chen^29^	2023	50	65	0	Sintilimab (PD-L1)	17.2	ORR
Rubinstein^27^	2023	67	0	100	Durvalumab (PD-L1)+Tremelimumab (CTLA-4)	6	PFS, ORR, CR, PR
Geurts^28^	2023	64.5	54	100	Durvalumab (PD-L1)	29	ORR, CR, PR
Qin^31^	2022	53	52.9	100	Serplulimab (PD-1)	7.7	ORR, PFS, OS
O’Malley^33^	2022	64	0	100	Pembrolizumab (PD-1)	42.6	ORR, PFS, OS
Bellone^39^	2022	69	0	100	Pembrolizumab (PD-1)	25.8	ORR, DCR, PFS, OS
Cercek^38^	2022	54	38	0	Dostarlimab (PD-1)	12	ORR
Oaknin^32^	2022	64.5	0	100	Dostarlimab (PD-1)	16.3	ORR, DOR, DCR
Diaz^37^	2022	63	46.4	14.4 (adjuvant therapy); 2 (neoadjuvant therapy)	Pembrolizumab (PD-1)	44.5	ORR, DCR, PFS, OS
Kwon^51^	2021	69	47.4	100	Pembrolizumab (PD-1)	19.5	PR, CR, SD, ORR, DCR, OS
Li^40^	2021	53	63.1	100	Envafolimab (PD-L1)	11.5	ORR, DOR, DCR, PFS, OS
Hollebecque^41^	2021	NR	51.2	100	LY3300054 (PD-L1), LY3321367 (anti-TIM-3 antibody)	LY3300054 (PD-L1):14.8; LY3321367 (anti-TIM-3 antibody):11.9	PR, CR, SD, ORR, PFS, OS
Chao^42^ (NCT02335411)	2021	62	85.7	100	Pembrolizumab (PD-1)	5.6	12-month OS, median PFS, ORR
Chao^42^ (KEYNOTE-061)	2021	67	46.7	100	Pembrolizumab (PD-1)	7.9	12-month OS, median PFS, ORR
Chao^42^ (KEYNOTE-062)	2021	62	50	0	Pembrolizumab (PD-1)	11.3	12-month OS, median PFS, ORR
Marabelle^54^	2020	60	41.2	97	Pembrolizumab (PD-1)	13.4	ORR, DOR
Le (Cohort A)^53^	2020	53	59	100	Pembrolizumab (PD-1)	31.3	ORR, DOR, DCR, PFS
Le (Cohort B)^53^	2020	59	52	100	Pembrolizumab (PD-1)	24.2	ORR, DOR, DCR, PFS
Cohen^43^	2020	56.5	52.6	100	Nivolumab (PD-1)+ipilimumab (CTLA-4)	18.1	DCR, PFS, OS
Azad^54^	2020	60	33	100	Nivolumab (PD-1)	17.3	ORR, 6-, 12-, and 18-month PFS
Konstantinopoulos^55^	2019	NR	0	100	Avelumab (PD-L1)	18.6	ORR, 6-month PFS
Michael^44^	2018	58	70	100	Nivolumab (PD-1)+ipilimumab (CTLA-4)	13.4	ORR, OS

DCR, disease control rate; DOR, duration of response; NA, not available; ORR, overall response rate.

### Risk of bias assessment and grading evidence

In evaluating the 18 non-RCTs in our sample utilizing the Methodological Index for Non-Randomized Studies (MINORS) criteria, the studies averaged a score of 13 out of 20, signaling moderate to high quality with some areas needing improvement, particularly concerning the prospective collection of data and the unbiased assessment of outcomes. For the 6 RCTs included in our pooled analysis, the evaluation of risk of bias using the Risk of Bias (ROB) tool yielded mixed results. These trials were noted to have some concerns in the ‘other bias’ domain due to concerns such as funding sources. Details of evaluations for the risk of bias are presented in Tables 2 and 3.

**Table 2:** Methodological quality of the included studies (MINORS).

Reference	Year	1^a^	2^b^	3^c^	4^d^	5^e^	6^f^	7^g^	8^h^	9^i^	10^j^	11^k^	12^l^	Total
André^50^ (GARNET)	2023	2	2	2	2	2	2	2	0	0	0	0	0	14
André^30^ (NEONIPIGA)	2023	2	2	2	2	0	2	2	0	0	0	0	0	12
Chen^29^	2023	2	2	2	2	1	2	2	0	0	0	0	0	13
Rubinstein^27^	2023	2	2	2	2	0	2	1	0	2	2	1	2	18
Geurts^28^	2023	2	2	2	2	1	2	2	0	0	0	0	0	13
Qin^31^	2022	2	2	2	2	1	2	2	0	0	0	0	0	13
O’Malley^33^	2022	2	2	2	2	1	2	2	0	0	0	0	0	13
Bellone^39^	2022	2	2	2	2	0	2	2	0	0	0	0	0	12
Cercek^38^	2022	2	2	2	2	0	2	2	0	0	0	0	0	12
Oaknin^32^	2022	2	2	2	2	1	2	2	0	0	0	0	0	13
Diaz^37^	2022	2	2	2	2	0	2	2	0	0	0	0	0	12
Kwon^51^	2021	2	2	2	2	0	2	2	0	0	0	0	0	12
Li^40^	2021	2	2	2	2	0	2	2	0	0	0	0	0	12
Hollebecque^41^	2021	2	2	2	2	0	2	2	0	2	2	2	2	20
Chao^42^ (NCT02335411)	2021	2	2	2	2	1	2	2	0	0	0	0	0	13
Chao^42^ (KEYNOTE-061)	2021	2	2	2	2	2	2	2	0	0	0	0	0	14
Chao^42^ (KEYNOTE-062)	2021	2	2	2	2	2	2	2	0	0	0	0	0	14
Marabelle^52^	2020	2	2	2	2	2	2	2	0	0	0	0	0	14
Le (Cohort A)^53^	2020	2	2	2	2	0	2	2	0	0	0	0	0	12
Le (Cohort B)^53^	2020	2	2	2	2	0	2	2	0	0	0	0	0	12
Cohen^43^	2020	2	2	2	2	1	2	2	0	0	0	0	0	13

^a^ A clearly stated aim.

^b^ Inclusion of consecutive patients.

^c^ Prospective collection of data.

^d^ Endpoints appropriate to the aim of the study.

^e^ Unbiased assessment of the study endpoint.

^f^ Follow-up period appropriate to the aim of the study.

^g^ Loss to follow-up less than 5%.

^h^ Prospective calculation of the study size.

^i^ An adequate control group.

^j^ Contemporary groups.

^k^ Baseline equivalence of groups.

^l^ Adequate statistical analyses.

**Table 3:** Methodological quality for randomized controlled trials (ROB).

Author	Year	1^a^	2^b^	3^c^	4^d^	5^e^	6^f^	7^g^
Westin^48^	2024	Low	Low	Low	Low	Low	Low	Unclear
Mirza^47^	2023	Low	Low	Low	Low	Low	Low	Unclear
Taïeb^49^	2023	Low	Low	Low	Low	Low	Low	Unclear
Eskander^46^	2023	Low	Low	Low	Low	Low	Low	Unclear
Diaz^37^	2022	Low	Low	Low	Low	Unclear	Low	Unclear
Chao^42^ (KEYNOTE-061)	2021	Low	Low	Low	Low	Low	Low	Unclear
Chao^42^ (KEYNOTE-062)	2021	Low	Low	Low	Low	Low	Low	Unclear

^a^ Random sequence generation (selection bias).

^b^ Allocation concealment (selection bias).

^c^ Blinding of participants and personnel (performance bias).

^d^ Blinding of outcome assessment (detection bias).

^e^ Incomplete outcome data (attrition bias).

^f^ Selective reporting (reporting bias).

^g^ Other bias.

The detailed GRADE evidence profiles, including justification for downgrading or upgrading the quality of evidence, have been provided in Supplementary Table S1 (Supplemental Digital Content 3, http://links.lww.com/JS9/D331). To summarize, the GRADE ratings indicated that the evidence for ORR, DCR, 12-month OS, and 12-month PFS rates was of moderate certainty, reflecting some concerns primarily related to inconsistency across studies. The evidence for 24-month OS, 36-month OS, 24-month PFS, and 36-month PFS rates ranged from moderate to low certainty, with some concerns related to inconsistency across studies and imprecision.

The kappa statistic for overall agreement across the processes of study inclusion, data extraction, risk of bias, and grading evidence evaluations was found to be 0.87, indicative of a high level of agreement exceeding chance, which confirms the robustness and reliability of our study evaluations.

### Primary analysis

**Figure 2:**
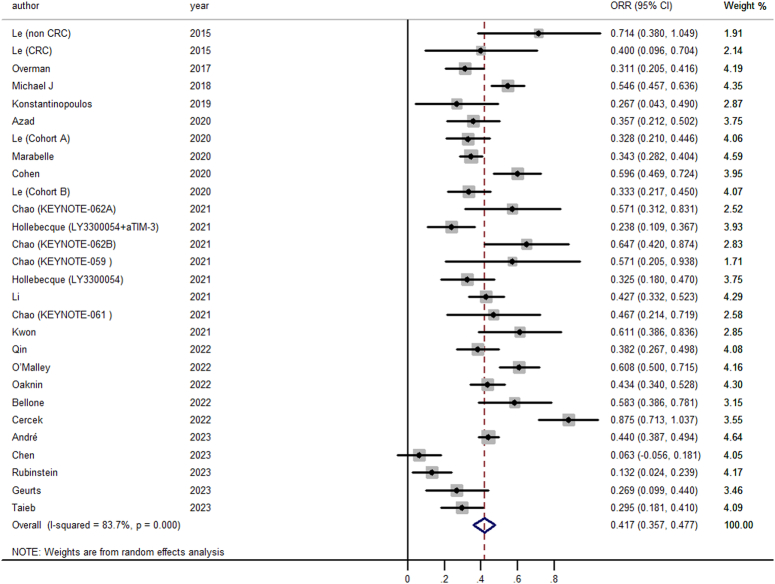
Forest plot for the pooled estimate of disease control rate (DCR).

The pooled estimate of ORR from the 23 studies revealed a notable 41.7% (95% CI, 35.7–47.7%) (Fig. 2), symbolizing a substantial antitumor effect. However, substantial heterogeneity was present (*I* ^2^=83.7%, *P*<0.01), prompting further investigation through subgroup analyses. Subgroup analysis, delineated in Table 4, targeted potential sources of variability, including trial phase, cancer type, and ICI class. The results underscored higher ORR in phase 3 trials (ORR=56.8%, 95% CI, 42.6–70.9%) compared to phase 1 (ORR=37.4%, 95% CI, 28.6–46.3%) and phase 2 (ORR=41.5%, 95% CI, 33.8–49.3%). Notably, studies on endometrial cancer exhibited an ORR of 43.0% (95% CI, 27.4–58.7%), while those on colorectal cancer reflected a slightly lower ORR of 39.5% (95% CI, 23.0–56.1%). Additionally, trials with PD-1+CTL-4 treatments reported an ORR of 56.3% (95% CI, 49.0–63.6%), surpassing those with PD-1 (ORR=43.9%, 95% CI, 35.3–52.5%) or PD-L1 (ORR=36.8%, 95% CI, 29.2–46.7%). To assess the potential impact of publication bias on our findings, funnel plot asymmetry was explored and substantiated by Egger’s test (*P*=0.427), suggesting no influence of publication bias. Sensitivity analyses conducted by iteratively removing each study confirmed the robustness of our pooled ORR, which remained stable across a range of 38.8–41.0%.

**Table 4:** Subgroup analyses for ORR.

Variables	ORR (95% CI)	*I* ^2^ (%)	Number of Studies	*P* for heterogeneity between subgroups
Sample size				0.025
<100	41.7 (33.4–50.0)	84.9	19	
≥100	43.4 (37.0–49.8)	72.0	5	
Study year				0.764
2015–2020	40.5 (32.7–48.3)	72.8	8	
2021–2022	42.6 (34.0–51.2)	87.1	16	
Location				0.780
Single country	41.7 (29.9–53.4)	91.1	11	
Multiple countries	41.5 (35.3–47.8)	70.0	13	
Study design				0.043
Phase 1	37.4 (28.6–46.3)	68.8	3	
Phase 2	41.5 (33.8–49.3)	86.5	19	
Phase 3	56.8 (42.6–70.9)	0	2	
Cancer type				0.045
Endometrial cancer	43.0 (27.4–58.7)	90.1	6	
Colorectal cancer	39.5 (23.0–56.1)	92.4	6	
Mixed cancer	39.6 (34.3–44.9)	50.2	11	
Mean/median age, years				0.233
<60	38.6 (29.6–47.5)	82.7	10	
≥60	44.6 (36.2–52.9)	87.2	14	
Sex				
Male	–	–	–	0.889
Female	40.5 (21.3–59.7)	90.8	5	
Mixed	41.8 (35.5–48.1)	81.9	19	
Pretreatment				0.770
Yes	40.0 (34.8–45.1)	75.8	21	
No	53.6 (10.1–97.0)	95.7	3	
ICI type				<0.001
PD-1	43.9 (35.3–52.5)	86.2	11	
PD-L1	36.8 (29.2–46.7)	44.5	9	
PD-1+CTL-4	56.3 (49.0–63.6)	0	2	
Mean/median follow-up period, months				0.085
<12	42.4 (31.7–53.2)	74.5	8	
≥12	41.4 (34.1–48.7)	86.6	16	

CI, confidence interval; ORR, overall response rate.

### Secondary analysis

**Figure 3:**
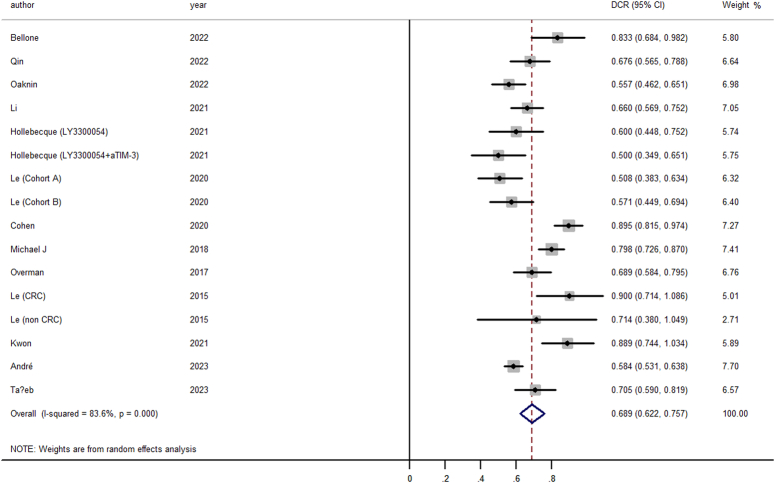
Forest plot for the pooled estimate of objective response rate (ORR).

In our secondary analysis, we delved into the DCR and key survival outcomes, including PFS and OS, across the aggregated study dataset. The pooled DCR stood at an impressive 68.9% (95% CI, 62.2–75.7%) (Fig. 3) indicating a strong benefit in terms of disease stabilization and control.

**Figure 4:**
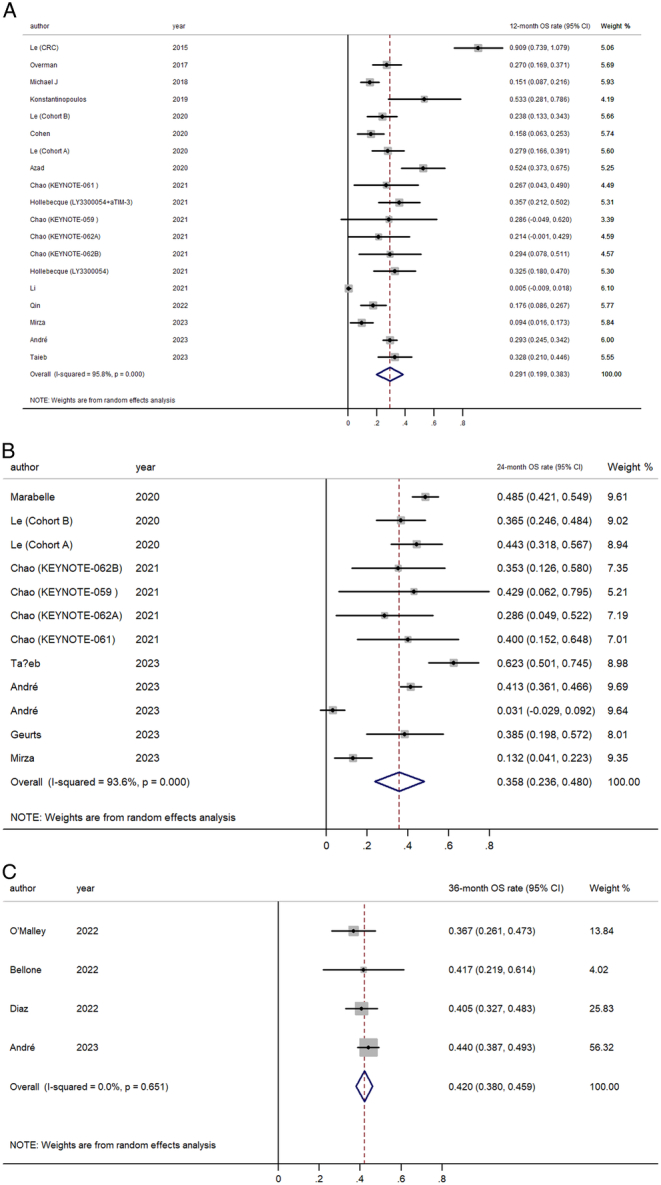
Forest plot for the pooled estimate of (A) 12-month overall survival (OS) rate; (B) 24-month OS rate; (C) 36-month OS rate.

**Figure 5:**
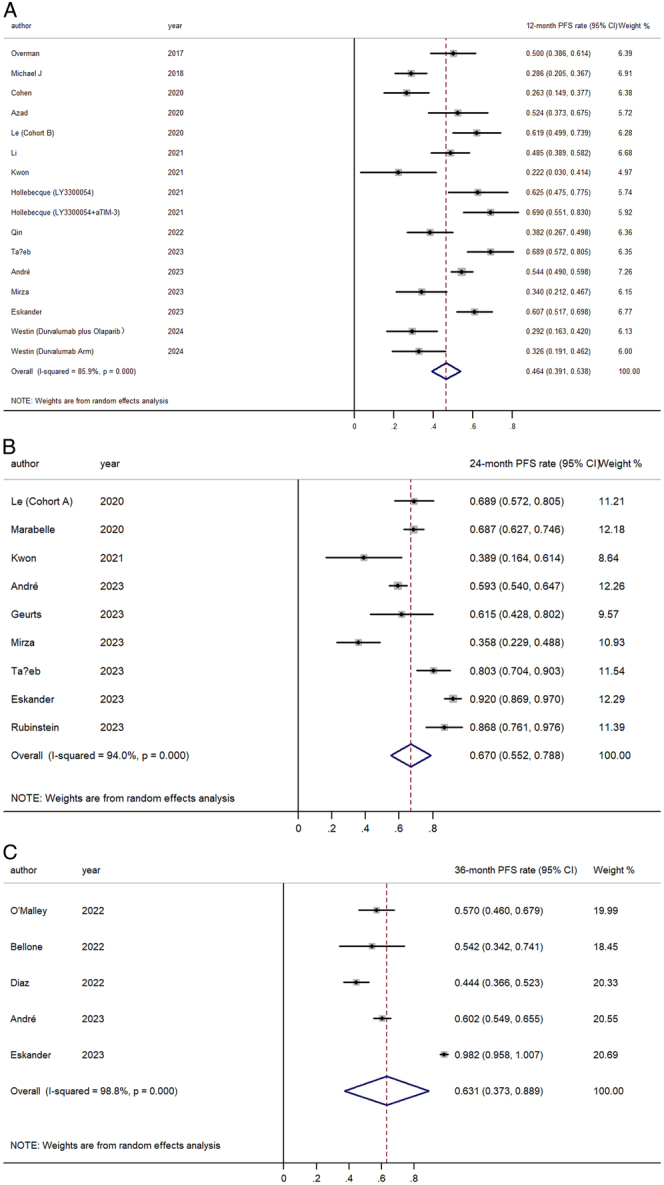
Forest plot for the pooled estimate of (A) 12-month progression-free survival (PFS) rate; (B) 24-month PFS rate; (C) 36-month PFS rate.

Exploring survival rates, we found that the pooled 12-month OS was 29.1% (95% CI, 19.9–38.3%), climbing to 35.8% (95% CI, 23.6–48.0%) at 24 months, and reaching 42.0% (95% CI, 38.0–45.9%) at the 36-month mark (Fig. 4). For PFS, the 12-month rate was observed to be at 46.4% (95% CI, 39.1–53.8%), with a notable increase to 67.0% (95% CI, 55.2–78.8%) at 24 months before a slight dip to 63.1% (95% CI, 37.3–88.9%), in the 36-month evaluation (Fig. 5).

Despite the compelling results, heterogeneity analysis indicated considerable variability with an *I* ^2^>50% across most of the OS and PFS timeframes, suggesting a diversity of treatment effects and patient populations. While the visual inspection of funnel plots revealed symmetry, both Begg’s and Egger’s tests were deployed to probe for publication bias, returning non-significant values (*P*>0.05), implying minimal influence of publication bias.

Sensitivity analysis, conducted by sequentially excluding individual studies, demonstrated that the pooled estimates for DCR, PFS, and OS remained within a relatively narrow confidence interval, showcasing the stability of our results despite the underlying heterogeneity.

## Discussion

### Principal findings of the study

Our comprehensive meta-analysis of phase I–III clinical trials evaluating immune ICIs in advanced cancers characterized by dMMR or MSI-H highlights encouraging therapeutic outcomes. The analysis indicates that patients harboring dMMR or MSI-H tumors demonstrate a robust response to ICIs, as substantiated by improved ORR, DCR, OS, and PFS. These findings are consistent across subgroups of studies with different designs, ICI types, and cancer types, with the hypothesis that the higher mutational burden in dMMR/MSI-H cancers increases the number of neoantigens, thereby enhancing the immunogenicity of the tumors and providing more targets for ICIs. Nevertheless, the comparative efficacy of ICIs between dMMR/MSI-H and proficient mismatch repair/microsatellite stable (pMMR/MSS) tumors remains to be definitively established and warrants further investigation.

### Potential mechanisms of the study

The superior efficacy of ICIs in dMMR/MSI-H tumors can be attributed to the increased immunogenicity resulting from the accumulation of neoantigens that arise from the hypermutation state^56,57^. These tumors are more likely to be recognized by the immune system as foreign, making them better candidates for ICIs that enhance immune activity^58,59^. Furthermore, the inflammatory tumor microenvironment, often associated with dMMR/MSI-H, may facilitate immune cell infiltration and activity upon ICI therapy^60^.

### Comparisons with similar meta-analyses

Our study distinguished itself from previously published works in several significant ways. While earlier studies have provided valuable insights into the clinical efficacy of ICIs in specific tumor types, such as colorectal and gastric cancers, these studies have been notably limited in their scope^64–66^. They have either focused exclusively on individual malignancies or employed mixed-method designs that included observational studies and phase 2 trials, which inherently lack the robustness and generalizability necessary to derive high-level evidence regarding the overall impact of ICIs across multiple tumor spectra^67,68^. Our study, by contrast, presents a comprehensive pooled analysis that consolidates quantitative data from phase I through III clinical trials, offering the most exhaustive evaluation to date on the clinical outcomes of ICI therapy in advanced cancers characterized by dMMR/MSI-H. Unlike prior meta-analyses, we have not confined our exploration to single entities but rather taken a pan-cancer approach, thus allowing us to systematically assess the therapeutic benefits of ICIs across a broad array of MSI-H advanced malignancies.

### Strengths of the study

Our study has several evident strengths. One of the foremost strengths of our research is the extensive compilation of data derived from a broad spectrum of clinical trials involving patients with dMMR/MSI-H advanced cancers. By integrating data across all phases from I to III, we have created a highly robust dataset that enables a thorough assessment of the clinical efficacy of ICIs, surpassing the limitations of narrower, single-tumor investigations. Secondly, the study adheres to strict methodological standards typical of high-quality meta-analyses. Our employment of rigorous statistical techniques ensures the validity and precision of our findings, reducing the likelihood of bias and increasing confidence in the conclusions drawn about the performance of ICIs in this complex and heterogeneous patient population. Thirdly, our study specifically focuses on dMMR/MSI-H status as predictive biomarkers for response to ICIs. This targeted approach allows for a deeper understanding of how these biomarkers influence treatment outcomes, offering oncologists tangible guidance for selecting appropriate therapies based on the genetic makeup of a patient’s tumor. Another key strength is the diversity of cancer types incorporated into the analysis. Unlike other studies that concentrate on isolated cancer entities, our pan-cancer perspective enhances the generalizability of the results, indicating that the therapeutic benefits observed might be applicable across multiple cancer types bearing the MSI-H/dMMR phenotype. Lastly, the inclusion criterion of only phase I–III clinical trials significantly elevates the quality of evidence underpinning our findings. These trials are typically characterized by stricter methodologies and control groups, which translates to higher-grade evidence compared to observational studies or early-phase trials. Thus, our study contributes to the highest level of scientific evidence currently available on the use of ICIs in the context of MSI-H/dMMR advanced cancers, solidifying its value in informing clinical practice and future research directions.

### Limitations of the study

We must acknowledge that several limitations exist in our study. Firstly, despite the rigorous methodology applied, one inherent limitation is the heterogeneity of data sourced from different clinical trials. Each trial has its unique design, eligibility criteria, treatment protocols, and follow-up periods, which could introduce variability into the combined analysis. This variation may affect the comparability of results across studies and limit the interpretation of pooled estimates regarding the effectiveness of immune checkpoint inhibitors (ICIs) in the MSI-H/dMMR setting. Secondly, a considerable challenge encountered in our study pertains to the immaturity of survival data in certain trials. Due to the dynamic nature of cancer treatments and the time required for long-term follow-ups, not all included studies had fully matured OS or PFS data. Consequently, our conclusions might be subject to change as more mature data become available, potentially impacting the assessment of the true therapeutic benefit provided by ICIs. Thirdly, there exists variability in the methods used to identify and report dMMR/MSI-H status among the included trials. Different laboratories and platforms can lead to discrepancies in the detection rate and classification of these molecular features, which might introduce bias into our pooled analysis. This inconsistency could affect the accuracy of our conclusions about the relationship between biomarker status and response to ICIs. Finally, although our study includes data from a diverse array of cancer types, it is constrained in its ability to conduct detailed subgroup analyses for each specific cancer type due to the smaller sample sizes within individual subgroups. This lack of granularity hampers the ability to draw definitive conclusions about the precise impact of ICIs on the efficacy and safety profiles across different cancer subpopulations with MSI-H/dMMR status.

### Values for clinical practice and policymakers

Understanding the enhanced responsiveness of dMMR/MSI-H tumors to ICIs can shape treatment protocols and decision-making in oncology. Clinicians can confidently consider ICIs as a viable first-line or subsequent treatment option in dMMR/MSI-H advanced cancers, especially when conventional therapies fail. The data supports the prioritization of genetic testing for dMMR/MSI-H in advanced cancers, which is essential for personalized medicine. Policymakers can advocate for broader access to genetic profiling and ICIs, enabling more patients to benefit from these significant therapeutic advances.

## Conclusions

This meta-analysis underscores the therapeutic efficacy of ICIs in the management of dMMR/MSI-H advanced cancers, supporting the potential utility of MSI status as an indicator of immunotherapy response. It emphasizes the remarkable therapeutic potential of ICIs for these specific malignancies and advocates for incorporating routine MSI testing into clinical practice, aligning with precision oncology strategies driven by genetic profiling. While advocating for policy adjustments to broaden access to genetic testing and ICIs to optimize patient outcomes, our findings necessitate cautious interpretation due to inherent limitations. Specifically, the absence of direct comparisons with pMMR/MSS tumors precludes definitive conclusions regarding MSI’s predictive power. To enhance understanding, future studies should focus on standardizing biomarker evaluation methodologies, comprehensively gathering survival data, and employing larger, prospective, and more homogenous patient cohorts to refine our comprehension of the precise impact and mechanisms of ICIs in the dMMR/MSI-H cancer setting.

## Ethical approval

No ethical approval and patient consent were required for all analyses based on literature research.

## Source of funding

This work was financially supported by the National Natural Science Foundation of China (No.82204716), Department of Science and Technology of Guangdong Province (No.2021A1515110773, 2022A1515220198), Guangzhou Basic and Applied Basic Research Foundation(No.2023A03J0759) and Science and Technology Planning Project of Guangdong Province (No. 2023B1212060063) and Guangzhou Municipal Science and Technology Bureau (No.202201011785), and the Traditional Chinese Medicine Bureau of Guangdong Province (No.20225012).

## Author contribution

W.W. and Z.M.: study concept and design; W.W., Z.M., Y.C., J.J., Y.Q., K.S., N.Z., G.B., Y.T., X.Z., and Z.J.: acquisition, analysis, or interpretation of data; Z.M., W.W., X.Z., and Z.J.: drafting of the manuscript; W.W., Z.M., X.Z., and Z.J.: statistical analysis; Z.M., W.W., X.Z., and Z.J.: study supervision. All authors were involved in the administrative, technical, or material support; also, the critical revision of the manuscript for important intellectual content. Lead contact: Zubing Mei.

## Conflicts of interest disclosure

The authors declare that they have no conflicts of interest.

## Research registration unique identifying number (UIN)

1. Name of the registry: PROSPERO (International Prospective Register of Systematic Reviews).
2. Unique identifying number or registration ID: CRD42024504419.
3. Hyperlink to your specific registration (must be publicly accessible and will be checked): not applicable.

## Guarantor

Zubing Mei, MD, PhD, Assistant Professor of Anorectal Disease Institute of Shuguang Hospital.

## Data availability statement

The data in this study is not sensitive in nature and is accessible in the public domain. The data is, therefore, available and not of a confidential nature. The datasets yielded during the study process are available from the corresponding author on reasonable request.

## Provenance and peer review

Not commissioned, externally peer-reviewed.

## Supplementary Material
