# Supplementary figures and images for: Immune checkpoint inhibitors for patients with mismatch repair deficient or microsatellite instability-high advanced cancers: a meta-analysis of phase I–III clinical trials
Source: Int J Surg. 2024 Aug 14;111(1):1357–72. doi: 10.1097/JS9.0000000000002007 (PMC11745646; doi:10.1097/JS9.0000000000002007)

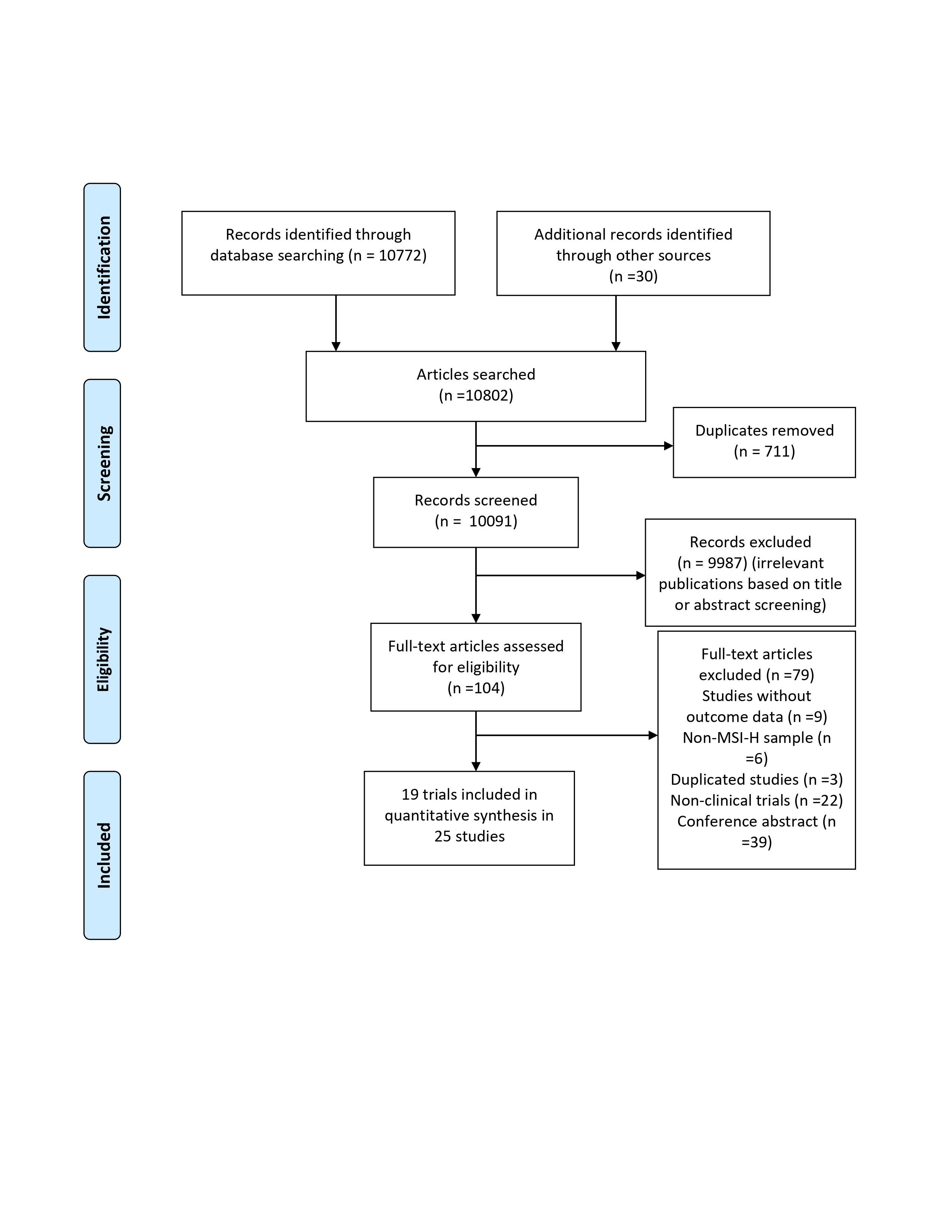

Supplement: Supplementary file 2 [file js9-111-1357-s002.jpg]
