# Supplementary material for: Immune checkpoint inhibitors for patients with mismatch repair deficient or microsatellite instability-high advanced cancers: a meta-analysis of phase I–III clinical trials
Source: Int J Surg. 2024 Aug 14;111(1):1357–72. doi: 10.1097/JS9.0000000000002007 (PMC11745646; doi:10.1097/JS9.0000000000002007)
Supplement: Supplementary file 3 [file js9-111-1357-s003.docx]

**Supplementary Material 1**

**Text S1 Search strategy**

**Database: Pubmed from inception to Present> (Search date: September 9, 2023 and updated in June 22, 2024)**

**Search Strategy:**

--------------------------------------------------------------------------------

***Cancer terms:***

1 "Neoplasms"[Mesh]

2 (cancer* or oncology* or tumor* or tumour* or neoplas* or malignan* or carcinoma* or lymphoma or adenocarcinoma* or melanoma or choriocarcinoma* or leukemia* or leukaemia* or metastat* or sarcoma* or teratoma*) [Title/Abstract]

3 or/1-2

***Immune Checkpoint Inhibitor terms:***

4. "Immune Checkpoint Inhibitors"[Mesh]

5. "Cell Cycle Checkpoints"[Mesh]

6 "CTLA-4 Antigen"[Mesh]

7 (check-point inhibitor* or checkpoint inhibitor* or CTLA-1 or CTLA-4 or cytotoxic T-lymphocyte-associated protein 4 or PD-L1 or PD-1 or programmed death receptor 1 or immune checkpoint inhibitor or ipilimumab or tremelimumab or nivolumab or pembrolizumab or durvalumab or atezolizumab or cemiplimab or spartalizumab or MED10680 OR AMP-224 OR pidilizumab OR atezolimab OR MED14736 OR avelumab OR BMS-936559 AND durvalumab OR MEDI4736) [Title/Abstract]

8 or/4-7

***Microsatellite Instability terms:***

9 "Microsatellite Instability"[Mesh]

10 "DNA Mismatch Repair"[Mesh]

11 ( microsatellite instability or MSI-H or MSI or mismatch-repair deficient or microsatellite repeats or mismatch repair or replication error or BAT25 or BAT26 or D5S346 or D2S123 or D17S250 or dMMR or MLH1 or MSH2 or MSH6 or PMS2) [Title/Abstract]

12 or/9-11

***Final search results: Combined:***

13 3 and 8 and 12

**Text S2 Search strategy**

**Database: EMBASE (Search date: September 9, 2023 and updated in June 22, 2024)**

**Search Strategy:**

--------------------------------------------------------------------------------

***Cancer terms:***

1 'neoplasm'/exp

2 (cancer* or oncology* or tumor* or tumour* or neoplas* or malignan* or carcinoma* or lymphoma or adenocarcinoma* or melanoma or choriocarcinoma* or leukemia* or leukaemia* or metastat* or sarcoma* or teratoma*):ab,ti

3 or/1-2

***Immune Checkpoint Inhibitor terms:***

4. 'immune checkpoint inhibitor'/exp

5. 'cell cycle checkpoint'/exp

6 'cytotoxic T lymphocyte antigen 4'/exp

7 (‘check-point inhibitor*’ or ‘checkpoint inhibitor*’ or CTLA-1 or CTLA-4 or ‘cytotoxic T-lymphocyte-associated protein 4’ or PD-L1 or PD-1 or ‘programmed death receptor 1’ or ‘immune checkpoint inhibitor’ or ipilimumab or tremelimumab or nivolumab or pembrolizumab or durvalumab or atezolizumab or cemiplimab or spartalizumab or MED10680 OR AMP-224 OR pidilizumab OR atezolimab OR MED14736 OR avelumab):ab,ti

8 or/4-7

***Microsatellite Instability terms:***

9 'microsatellite instability'/exp

10 'mismatch repair'/exp

11 (‘microsatellite instability’ or ‘mismatch-repair deficient’ or ‘microsatellite repeats’ or ‘mismatch repair’ or ‘replication error’ or BAT25 or BAT26 or D5S346 or D2S123 or D17S250 or dMMR or MLH1 or MSH2 or MSH6 or PMS2 or MSI-H or MSI) :ab,ti

12 or/9-11

***Final search results: Combined:***

13 3 and 8 and 12

**Text S3 Search strategy**

**Database: Cochrane Library from inception to Present> (Search date: September 9, 2023 and updated in June 22, 2024)**

**Search Strategy:**

--------------------------------------------------------------------------------

***Cancer terms:***

1 MeSH descriptor: [Neoplasms] explode all trees

2 (cancer* or oncology* or tumor* or tumour* or neoplas* or malignan* or carcinoma* or lymphoma or adenocarcinoma* or melanoma or choriocarcinoma* or leukemia* or leukaemia* or metastat* or sarcoma* or teratoma*):ti,ab,kw (Word variations have been searched)

3 1 or 2

***Immune Checkpoint Inhibitor terms:***

4 MeSH descriptor: [Immune Checkpoint Inhibitors] explode all trees

5 MeSH descriptor: [Cell Cycle Checkpoints] explode all trees

6 MeSH descriptor: [CTLA-4 Antigen] explode all trees

7 (check-point inhibitor* or checkpoint inhibitor* or CTLA-1 or CTLA-4 or cytotoxic T-lymphocyte-associated protein 4 or PD-L1 or PD-1 or programmed death receptor 1 or immune checkpoint inhibitor or ipilimumab or tremelimumab or nivolumab or pembrolizumab or durvalumab or atezolizumab or cemiplimab or spartalizumab or MED10680 OR AMP-224 OR pidilizumab OR atezolimab OR MED14736 OR avelumab OR BMS-936559 AND durvalumab OR MEDI4736):ti,ab,kw (Word variations have been searched)

8 4 or 5 or 6 or 7

***Microsatellite Instability terms:***

9 MeSH descriptor: [Microsatellite Instability] explode all trees

10 MeSH descriptor: [DNA Mismatch Repair] explode all trees

11 (microsatellite instability or MSI-H or MSI or mismatch-repair deficient or microsatellite repeats or mismatch repair or replication error or BAT25 or BAT26 or D5S346 or D2S123 or D17S250 or dMMR or MLH1 or MSH2 or MSH6 or PMS2):ti,ab,kw (Word variations have been searched)

12 9 or 10 or 11

***Final search results: Combined:***

13 3 and 8 and 12

**Text S4 Search strategy**

**Database: Google Scholar from inception to Present> (Search date: June 22, 2024)**

**Search Strategy:**

--------------------------------------------------------------------------------

1. Cancer
2. Immune Checkpoint Inhibitor
3. Microsatellite Instability
4. 1 AND 2 AND 3

**Supplementary Table 1.** GRADE Evidence Summary

| **Outcome Measure** | **Initial Quality Rating** | **Downgrading/Upgrading Factors** | **Final GRADE** |
| --- | --- | --- | --- |
| Objective Response Rate (ORR) | Moderate (mixed designs) | - Inconsistency: High I² (downgrade)  - Imprecision: Low due to narrow CI width  - Low risk of bias and publication bias (no downgrade)  - No upgrading factors noted | Moderate |
| Disease Control Rate (DCR) | Moderate (mixed designs) | - Inconsistency: High I² (downgrade)  - Imprecision: Low due to narrow CI width  - Low risk of bias and publication bias (no downgrade)  - No upgrading factors noted | Moderate |
| 12-month Overall Survival (OS) | Moderate (mixed designs) | - Inconsistency: High I² (downgrade)  - Imprecision: Low due to narrow CI width  - Low risk of bias and publication bias (no downgrade)  - No upgrading factors noted | Moderate |
| 24-month OS | Moderate (mixed designs) | - Inconsistency: High I² (downgrade)  - Imprecision: Moderate to substantial (downgrade)  - Low risk of bias and publication bias (no downgrade)  - No upgrading factors noted | Low |
| 36-month OS | Moderate (mixed designs) | - Inconsistency: Very low (no downgrade)  - Imprecision: Moderate due to CI width (downgrade considered)  - Low risk of bias and publication bias (no downgrade)  - No upgrading factors noted | Low |
| 12-month Progression-Free Survival (PFS) | Moderate (mixed designs) | - Inconsistency: High I² (downgrade)  - Imprecision: Low due to narrow CI width  - Low risk of bias and publication bias (no downgrade)  - No upgrading factors noted | Moderate |
| 24-month PFS | Moderate (mixed designs) | - Inconsistency: High I² (downgrade)  - Imprecision: Moderate (downgrade considered)  - Low risk of bias and publication bias (no downgrade)  - No upgrading factors noted | Low |
| 36-month PFS | Moderate (mixed designs) | - Inconsistency: High I² (downgrade)  - Imprecision: Substantial (downgrade)  - Low risk of bias and publication bias (no downgrade)  - No upgrading factors noted | Low |

Note: The GRADE system evaluates evidence across five key domains: study design, risk of bias, inconsistency, indirectness, imprecision, and publication bias, with potential upgrading factors also considered. Below is a summary of the GRADE assessment for each outcome, noting that the initial grading for RCTs is typically 'high', but can be downgraded based on the criteria mentioned.
